# Low-dose DNA demethylating therapy induces reprogramming of diverse cancer-related pathways at the single-cell level

**DOI:** 10.1186/s13148-020-00937-y

**Published:** 2020-09-21

**Authors:** Hideyuki Takeshima, Yukie Yoda, Mika Wakabayashi, Naoko Hattori, Satoshi Yamashita, Toshikazu Ushijima

**Affiliations:** 1 Division of Epigenomics, National Cancer Center Research Institute, Tokyo, Japan; 2 Department of Surgery, Faculty of Medicine, Saga University, Saga, Japan

**Keywords:** Epigenetics, DNA methylation, Epigenetic therapy, Single cell analysis

## Abstract

**Background:**

Epigenetic reprogramming using DNA demethylating drugs is a promising approach for cancer therapy, but its efficacy is highly dependent on the dosing regimen. Low-dose treatment for a prolonged period shows a remarkable therapeutic efficacy, despite its small demethylating effect. Here, we aimed to explore the mechanisms of how such low-dose treatment shows this remarkable efficacy by focusing on epigenetic reprograming at the single-cell level.

**Methods:**

Expression profiles in HCT116 cells treated with decitabine (DAC) were analyzed by single-cell RNA-sequencing (scRNA-seq). Functional consequences and DNA demethylation at the single-cell level were analyzed using cloned HCT116 cells after DAC treatment.

**Results:**

scRNA-seq revealed that DAC-treated cells had highly diverse expression profiles at the single-cell level, and tumor-suppressor genes, endogenous retroviruses, and interferon-stimulated genes were upregulated in random fractions of cells. DNA methylation analysis of cloned HCT116 cells revealed that, while only partial reduction of DNA methylation levels was observed in bulk cells, complete demethylation of specific cancer-related genes, such as cell cycle regulation, WNT pathway, p53 pathway, and TGF-β pathway, was observed, depending upon clones. Functionally, a clone with complete demethylation of *CDKN2A* (*p16*) had a larger fraction of cells with tetraploid than parental cells, indicating induction of cellular senescence due to normalization of cell cycle regulation.

**Conclusions:**

Epigenetic reprogramming of specific cancer-related pathways at the single-cell level is likely to underlie the remarkable efficacy of low-dose DNA demethylating therapy.

## Introduction

Epigenetic alterations, represented by aberrant DNA methylation, are frequently observed in various types of cancers [1, 2]. A wide range of tumor-suppressor genes, such as *CDH1*, *CDKN2A* (*p16*), and *MLH1*, are silenced by aberrant methylation of their promoter CpG islands (CGIs). These methylation-silenced genes are promising targets for cancer therapy, especially cancers with a limited number of driver mutations. DNA demethylating drugs, 5-azacytidine (azacytidine) and 5-aza-2′-deoxycytidine (decitabine, DAC), are now clinically used for patients with myelodysplastic syndrome (MDS) and acute myeloid leukemia (AML) [3–5]. Also in solid tumors, clinical trials using these demethylating drugs are being actively conducted [6, 7], and efficacy has already been shown for various types of solid tumors, such as recurrent metastatic non-small cell lung cancer [8], platinum-resistant ovarian cancers [9], advanced hepatocellular carcinomas [10], and drug-resistant relapsed/refractory alimentary tract cancers [11].

Early clinical studies used DNA demethylating drugs with the maximum tolerated dose (MTD) aiming at cytotoxic effects, and such studies showed only limited therapeutic efficacy with strong toxicity [12]. Afterwards, administration with a dose much lower than the MTD for a prolonged period was shown to have high therapeutic efficacy with limited toxicity [13–15]. The therapeutic effect was first explained by re-activation of a specific tumor-suppressor gene, such as *CDKN2A* [16], and was then shown to be associated with the suppression of tumor-initiating cells by restoration of multiple pathways in tumor cells [17]. In addition, enhancement of antigenicity of tumor cells by activation of endogenous retroviruses [18, 19] was found to be an important mode of action. Recently, in addition to the effect on tumor cells, that on tumor cell niche, including cancer-associated fibroblasts and myeloid-derived suppressor cells (MDSCs) has been suggested also to be involved [20–22].

Despite the remarkable therapeutic efficacy of low-dose and prolonged treatment with reprograming of multiple target genes, one remaining question is why only partial demethylation of the target genes [15, 17] can exert such high therapeutic efficacy. Considering that cells have two alleles for most genes, it is expected that, at the single-cell level, demethylation of a specific gene should be complete, 50%, or none. In this study, we aimed to explore whether complete demethylation of specific genes is really induced at the single-cell level and to analyze the functional consequences of such complete demethylation of specific genes.

## Results

### DAC-treated single cells had highly diverse expression profiles

**Fig. 1:**
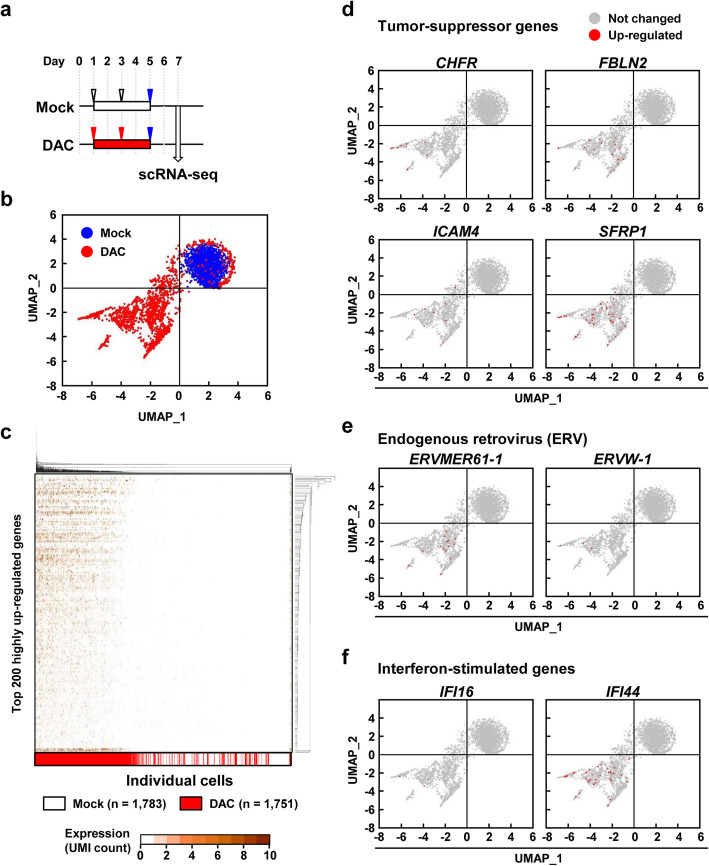
scRNA-seq of DAC-treated HCT116 cells. **a** Experimental protocol of DAC treatment. HCT116 cells were seeded on day 0 and treated with 0.5 μM of DAC on days 1 and 3. The cells were placed in fresh medium without DAC on day 5, and harvested on day 7. **b** UMAP using 14,099 genes that can be induced by DAC treatment. DAC-treated HCT116 cells had diverse expression profiles at the single-cell level. Blue dots, mock-treated cells. Red dots, DAC-treated cells. **c** Unsupervised hierarchical clustering analysis using the top 200 highly upregulated genes. Genes with higher expression levels were different, depending upon DAC-treated clones. White, mock-treated cells. Red, DAC-treated cells. **d**, **e**, and **f** Upregulation of specific tumor-suppressor genes, ERVs, and interferon-stimulated genes. Tumor-suppressor gene methylation silenced in colorectal cancers (*CHFR*, *FBLN2*, *ICAM4*, and *SFRP1*), ERVs (*ERVMER61-1* and *ERVW-1*), and interferon-stimulated genes (*IFI16* and *IFI44*) were upregulated in random fractions of cells

Single cell RNA sequencing (scRNA-seq) was conducted using 1783 mock-treated and 1751 DAC-treated HCT116 cells (Fig. 1a). On average, expression of 4867 and 5838 genes per cell was detected in mock- and DAC-treated cells, respectively. Uniform Manifold Approximation and Projection (UMAP) analysis was conducted using 14,099 genes that can be induced by DAC treatment (UMI counts ≤ 2 in all the 1783 mock-treated cells). It was shown that expression profiles in DAC-treated cells had high diversity (Fig. 1b). Hierarchical clustering analysis was conducted using highly upregulated genes (top 200 genes with higher mean UMI counts in DAC-treated single cells) selected from the 14,099 genes. It was shown that genes with higher expression levels were different, depending upon DAC-treated clones (Fig. 1c). Among the 1751 DAC-treated single cells, random fractions of cells showed upregulation (UMI counts ≥ 3) of specific established tumor-suppressor genes methylation-silenced in colorectal cancers [23–25] (*CHFR*, *FBLN2*, *ICAM4*, and *SFRP1*), endogenous retroviruses (ERVs) (*ERVMER61-1* and *ERVW-1*), and interferon-stimulated genes (*IFI16* and *IFI44*) (Fig. 1d, e, f). These results showed that DAC-treated single cells had diverse expression profiles.

### DAC-treated single cells exhibited complete DNA demethylation of specific CGIs

**Fig. 2:**
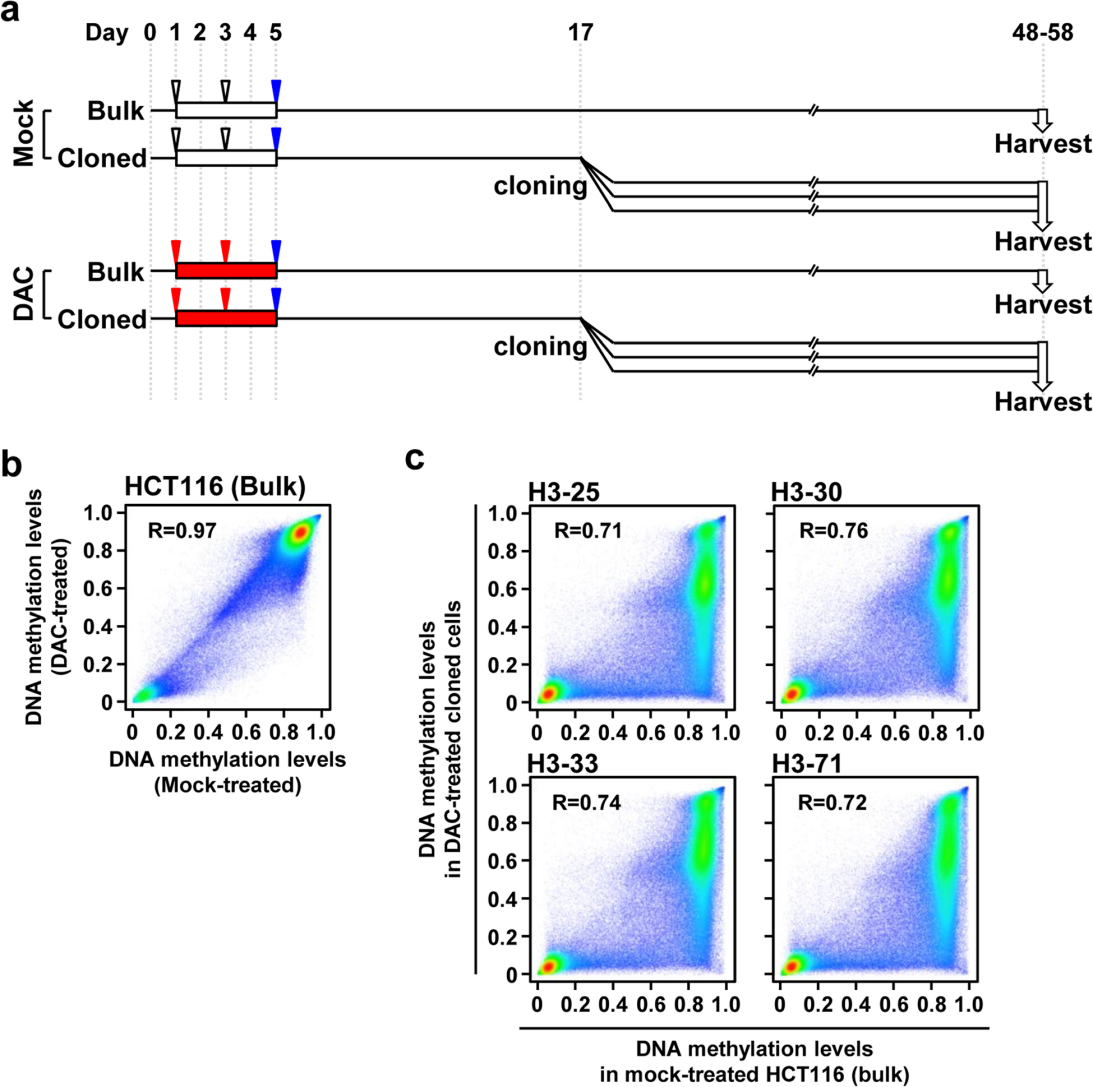
DNA methylation analysis of DAC-treated HCT116 cells at the single-cell level. **a** Experimental protocol of the single cell cloning of DAC-treated HCT116 cells. Single cells were isolated by the limiting dilution method, and nine clones were obtained. **b** Changes of DNA methylation levels in DAC-treated bulk cells. Only partial demethylation was induced by DAC treatment. **c** Changes of DNA methylation levels in DAC-treated clones. Methylation levels were markedly reduced by DAC treatment, and 12.1–28.2% of TSS200CGIs methylated in HCT116 cells were completely demethylated

To analyze the functional consequences of the diverse expression profiles and DNA methylation at the single-cell level, DAC-treated HCT116 cells were cloned by the limiting dilution method, and nine clones were obtained (Fig. 2a). Using these nine clones and their parental cells in bulk, DNA methylation was analyzed in a genome-wide manner, and methylation levels of 270,249 genomic blocks in DAC-treated bulk cells and clones were compared with those in mock-treated bulk cells. In the bulk cells, only partial reduction of DNA methylation levels was induced by DAC treatment (Fig. 2b, Fig. S1), and none of the 1039 TSS200CGIs (200 bp upstream regions from TSSs with CGIs) fully methylated in HCT116 cells were completely demethylated (*β* < 0.2). In contrast, in all of the clones, methylation levels were markedly reduced by DAC treatment (Fig. 2c, Fig. S2), and 126-293 of the 1039 TSS200CGIs were completely demethylated. These results showed that DAC-treated single cells exhibited complete demethylation of a large number of CGIs.

### Demethylated genes were highly diverse among the DAC-treated clones

**Fig. 3:**
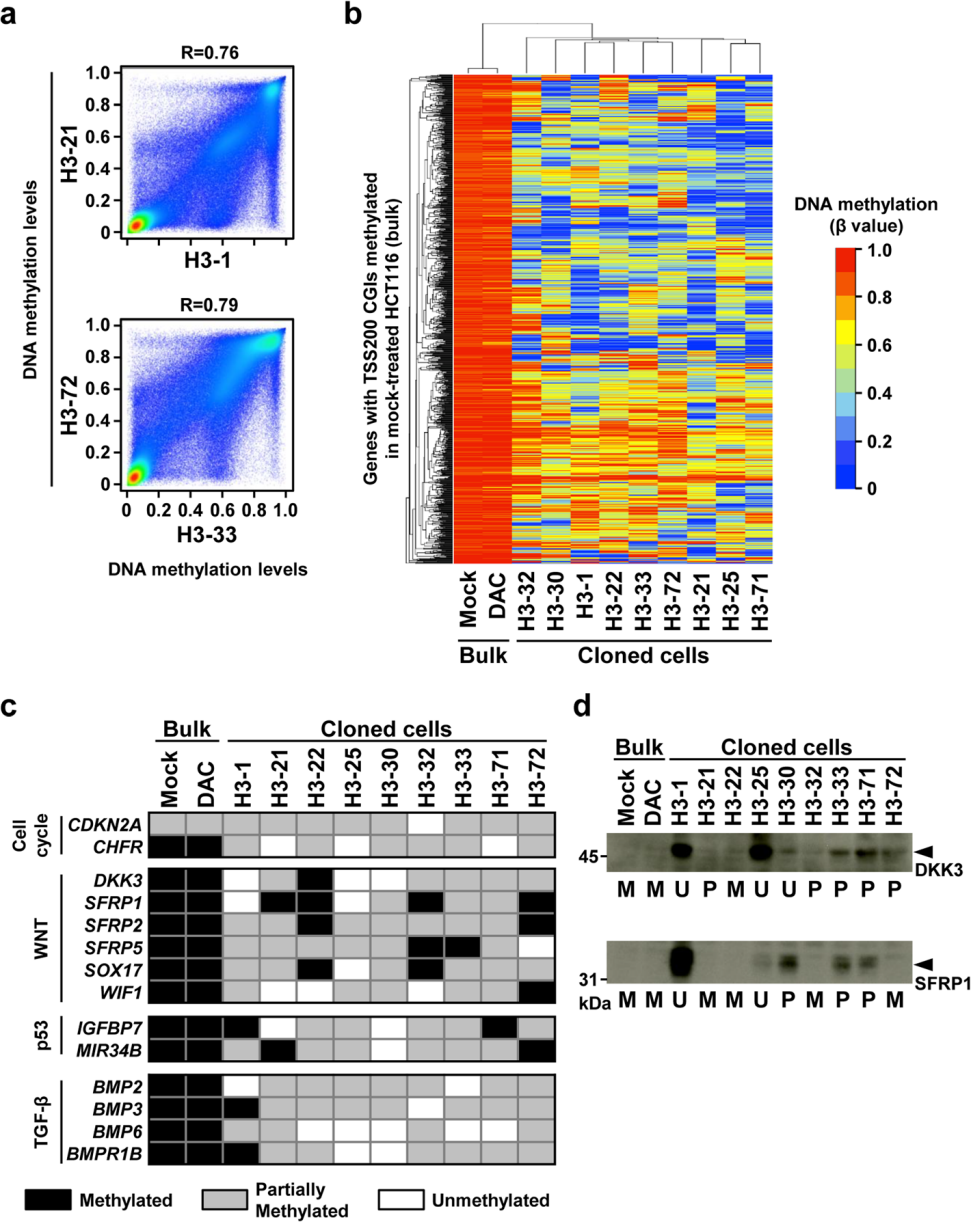
Difference of demethylated genes between DAC-treated clones. **a** Comparison of DNA methylation levels between DAC-treated clones. A large fraction of genomic blocks showed different methylation levels between the two clones compared. **b** Unsupervised hierarchical clustering analysis using DNA methylation profiles of 1039 TSS200CGIs fully methylated in HCT116. Demethylated genes were highly diverse among the DAC-treated clones. **c** DNA methylation status of genes involved in colon cancer-related pathways. Genes involved in cell cycle regulation, the WNT pathway, the p53 pathway, and the TGF-β pathway were completely demethylated, depending upon clones. **d** Protein expression levels of DKK3 and SFRP1 in DAC-treated clones. Complete demethylation led to the upregulation of methylation-silenced genes at the protein level in DAC-treated clones

To examine whether similar or different sets of genes were demethylated in different clones, DNA methylation levels of all the 270,249 genomic blocks in individual clones were compared. A large fraction of blocks showed different methylation levels among clones (Fig. 3a). DNA methylation status was then compared among clones for 1039 TSS200CGIs fully methylated in HCT116. Completely demethylated TSS200CGIs were highly diverse among the nine clones (Fig. 3b). Only 15.2–54.1% of completely demethylated TSS200CGIs overlapped (Table S1), and 700 (67.4%) of the 1039 TSS200CGIs were completely demethylated in at least one of the nine clones. These results showed that completely demethylated genes were highly diverse among the DAC-treated clones.

We next focused on genes involved in colon cancer-related pathways, namely cell cycle regulation, WNT pathway, p53 pathway, and TGF-β pathway. Regarding the cell cycle regulation, *CDKN2A* and *CHFR* were partially and fully methylated in HCT116, respectively. *CDKN2A* and *CHFR* were completely demethylated in one and three of the nine clones, respectively (Fig. 3c). Regarding the WNT pathway, six of its negative regulators, *DKK3*, *SFRP1*, *SFRP2*, *SFRP5*, *SOX17*, and *WIF1*, were fully methylated in HCT116. None of the six genes were demethylated in the DAC-treated bulk cells, but five of the six genes were completely demethylated in 1–3 of the nine clones (Fig. 3c). Genes involved in the p53 pathway, *IGFBP7* and *MIR34B*, and the TGF-β pathway, *BMP2*, *BMP3*, *BMP6*, and *BMPR1B*, were also completely demethylated, depending upon clones. It was confirmed that complete demethylation led to the upregulation of methylation-silenced genes at the protein level (Fig. 3d). These results showed that different sets of genes in different cancer-related pathways were completely demethylated at the single-cell level.

### Complete demethylation of specific genes induces normalization of specific cancer-related pathways

**Fig. 4:**
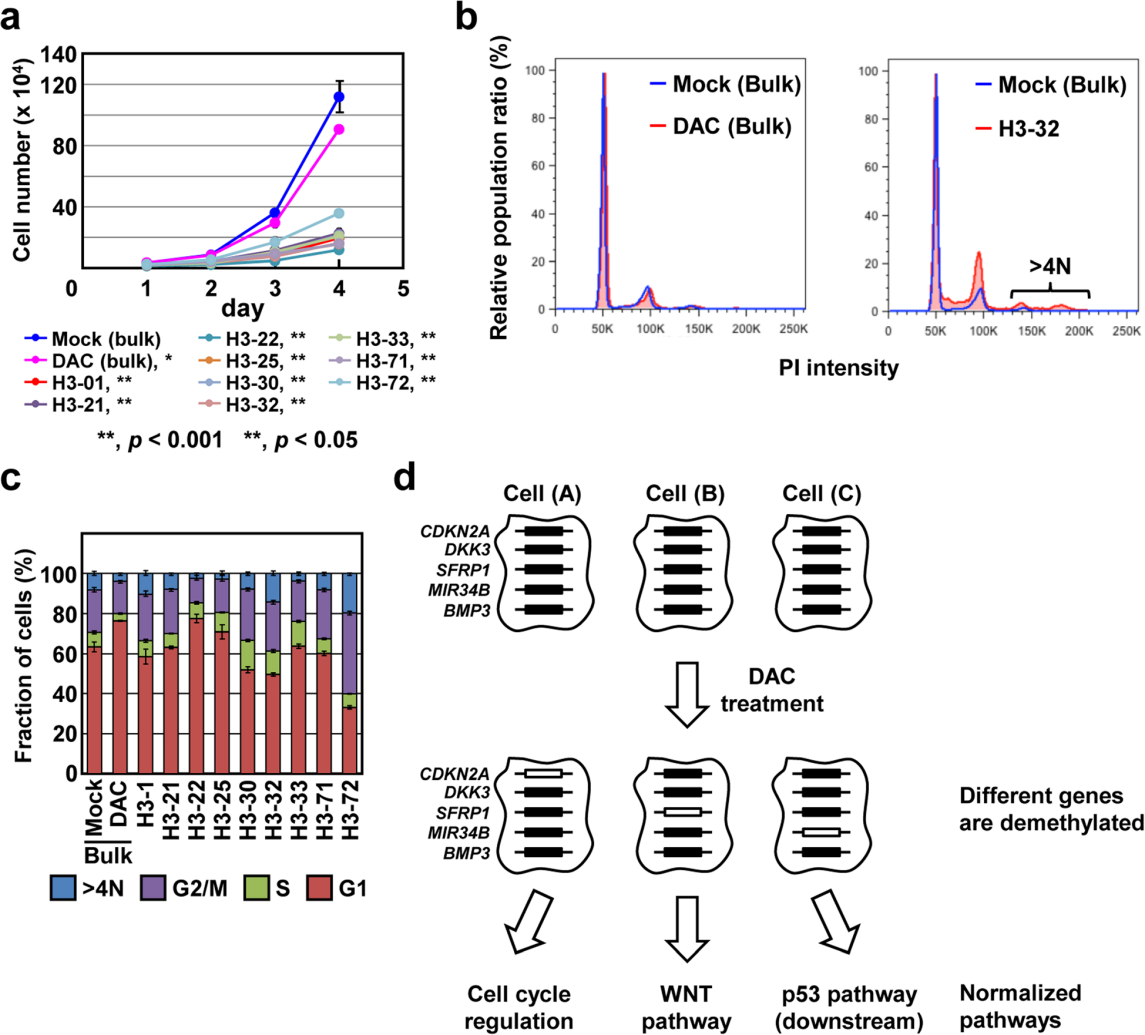
Analysis of a specific cancer-related pathway in DAC-treated clones. **a** Cell growth rates of DAC-treated clones. The growth rates of the nine clones were slower than those of the mock-treated and DAC-treated bulk cells, but the decreases were different depending upon the clones. **b** Cell cycle analysis of DAC-treated H3-32 clone. Cells with tetraploid were detected in the H3-32 clone, which had complete demethylation of *CDKN2A*, but not in the DAC-treated bulk cells. **c** Fraction of cells in specific phases of cell cycle. The H3-32 clone had a fraction of tetraploid cells much larger than that of mock-treated bulk cells. Data represent mean ± SD of triplicate experiments. **d** The model of the mechanism underlying therapeutic efficacy of low-dose demethylating therapy. Complete demethylation of a specific gene is induced at the single-cell level by demethylating therapy, and this leads to normalization of a specific cancer-related pathway, such as cell cycle regulation, the WNT pathway, and the p53 pathway

Changes of cell characteristics were first analyzed by examining cell proliferation of the nine clones along with mock-treated and DAC-treated bulk cells. The growth rates of the nine clones were slower than that of their parental cells, but the decreases were different among the clones (51.4–80.2%) (Fig. 4a). The doubling time of the individual clone was not associated with the number of completely demethylated genes (Fig. S3). These results suggested the diverse sets of re-activated genes, rather than the number of completely demethylated genes, led to diverse functional consequences.

To confirm that complete demethylation of a specific gene in a clone actually resulted in normalization of a specific cancer-related pathway, cell cycles in the DAC-treated clones, along with the DAC-treated bulk cells, were compared to that of mock-treated bulk cells. The H3-32 clone, which exhibited complete demethylation of *CDKN2A*, had a fraction of tetraploid (> 4 N) cells much larger than the mock-treated bulk cells (*p* = 0.0047) (Fig. 4b, c). In contrast, the seven of the other eight clones that did not exhibit complete demethylation, along with the DAC-treated bulk cells, did not have an increased fraction of tetraploid cells compared to the mock-treated bulk cells. Since expression of p16, encoded by *CDKN2A*, is known to play a key role in the induction of cellular senescence [26, 27], the increased fraction of tetraploid cells was considered to be due to the induction of cellular senescence by *CDKN2A* re-activation. This result showed that complete demethylation of a specific gene actually induced normalization of a specific cancer-related pathway.

## Discussion

Single-cell analysis revealed that diverse expression profiles were produced by a low-dose treatment with a DNA demethylating agent, here DAC. Different sets of genes in different cancer-related pathways were completely demethylated in individual cells, and such complete demethylation induced normalization of a specific cancer-related pathway, such as regulation of cell cycle. The finding solved the long-standing question, at least in part, of why low-dose DAC treatment can have strong therapeutic effects despite the fact that only partial demethylation is induced in bulk cells. Epigenetic reprogramming of different cancer-related pathways at the single-cell level, namely heterogeneous responses of individual cells to DAC, is likely to underlie the remarkable efficacy of low-dose DNA demethylating therapy (Fig. 4d). The heterogeneous response is considered to be due to the heterogeneous allelic demethylation. Based on these findings, it is considered that detection of only partial demethylation, but not complete demethylation, as a whole is sufficient to obtain therapeutic efficacy.

Usual single-cell analysis can provide vivid pictures of cellular heterogeneity even among cells that appear to be homogeneous. Here, to analyze functional consequences of the diverse expression profiles among DAC-treated cells, we combined the method of single-cell cloning with single-cell RNA-seq analysis. DNA methylation analysis and phenotypic analysis using cloned cells clearly showed normalization of cancer-related pathways at the single-cell level due to complete DNA demethylation and diversity in functional consequences. Acceleration of the step of single-cell cloning by additional innovation would further advance research addressing complex groups of cells without morphological differences.

Epigenetic reprograming of diverse pathways in individual cells could theoretically lead to production of more aggressive cells. However, in a clinical setting, good response is often observed, and cells in bulk show slower growth and less aggressive phenotypes [17]. In this study, the growth rates of all the nine DAC-treated clones were indeed slower than that of mock-treated and DAC-treated bulk cells, and clones with increased growth rates did not appear. As a possible mechanism of why only clones with less aggressive phenotypes are produced despite complete demethylation of various sets of genes at the single-cell level, we can note that cancer cells have already been highly selected as the fittest for multiple environments, and it is very difficult to reprogram so that the cancer cells would become even more aggressive.

The normalization of cellular functions in diverse directions by a DNA demethylating drug suggests that epigenetic therapy would be better combined with drugs with broad actions, such as cytotoxic drugs, but not with molecular targeted drugs. Indeed, DNA demethylating drugs were frequently combined with various types of cytotoxic drugs, such as cisplatin, temozolomide, and docetaxel, in many clinical trials [6, 28–30], but with molecular targeted drugs, such as panitumumab and erlotinib, in only a limited number of trials [31, 32]. Higher overall response has been achieved in combination therapy with cytotoxic drugs [6].

Surviving cells after DAC treatment, namely DAC-treated clones, were used for analysis of normalization of specific cancer-related pathways. The surviving cells were only partially demethylated as the dead cells (Fig. S1) but had 7.8% less demethylation based upon the mean of the changes of the *β* values in all genomic blocks. The 7.8% increased demethylation in dead cells was considered to lead to an increase of completely demethylated pathways to a similar degree. However, although some bias for the normalized pathways and their numbers may have existed, the concept of normalization of different sets of pathways in different cells is likely to be valid even in the dead cells. Unfortunately, dead cells cannot be used for single cell RNA-seq analysis or analysis of cellular functions, and this could be a limitation of this study.

## Conclusions

Different sets of genes in different cancer-related pathways were completely demethylated at the single-cell level, and this is likely to underlie the remarkable efficacy of low-dose DNA demethylating therapy.

## Materials and methods

### Single cell RNA sequencing

A human colon cancer cell line, HCT116, was purchased from the American Type Culture Collection (Rockville, MD). The absence of *Mycoplasma* infection was confirmed using the MycoAlert mycoplasma detection kit (Lonza, Basel, Switzerland). The cells (1 × 10^5^) were seeded on day 0 and treated with 0.5 μM of DAC (Sigma-Aldrich, St. Louis, MO) on days 1 and 3. The cells were placed in fresh medium without DAC on day 5 and harvested on day 7. With DAC treatment between 0.005 to 5 μM, the strongest DNA demethylation was observed with 0.5 μM of treatment (Fig. S4).

Using 1000 live cells of DAC-treated or mock-treated HCT116, a library was prepared using the Chromium Controller (10x Genomics, Pleasanton, CA) and Chromium Single Cell 3′ GEM, Library & Gel Bead Kit v3 (10x Genomics). The prepared scRNA-seq libraries were sequenced using an Illumina HiSeq platform (Illumina, San Diego, CA), and then, obtained sequence reads were de-multiplexed and mapped to the human genome (build hg38) using the Cell Ranger software (10x Genomics, version 3.0.2).

### Cluster analysis of scRNA-seq data

Dimension reduction and visualization of scRNA-seq data were conducted for 14,099 genes with UMI counts of 2 or less in all the mock-treated single cells by UMAP [33] (URL; https://arxiv.org/abs/1802.03426) using the R version 3.6.2 with the umap package. Hierarchical clustering analysis was conducted using the top 200 highly upregulated genes (top 200 genes with higher mean UMI counts in DAC-treated single cells) selected from the 14,099 genes using R function hclust (https://cran.r-project.org/).

### Cloning of HCT116 cells after DAC treatment

HCT116 cells (3 × 10^5^) were seeded on day 0 and treated with 0.5 μM of DAC on days 1 and 3, and the cells were placed in a fresh medium without DAC on day 5. The cells were cloned by the limiting dilution method on day 17 (DAC clones), and clones were harvested on days 48–58.

### Genome-wide DNA methylation analysis by BeadChip array

Genome-wide analysis of DNA methylation status was performed using an Infinium HumanMethylation450 BeadChip array as described [34]. The methylation level of each CpG site was represented by a *β* value that ranged from 0 (unmethylated) to 1 (fully methylated). All the 485,512 probes on the array were assembled into 279,922 genomic blocks based on their location against a transcription start site (TSS) and relative location against a CGI [35]. Among them, 7839 were located within TSS200CGIs. The DNA methylation level of a genomic block was evaluated using the mean *β* value of all the probes within the genomic block, and the methylation status of the genomic block was classified into unmethylated (*β* value < 0.2), partially methylated (0.2 ≤ *β* value < 0.8), and fully methylated (0.8 ≤ *β* value). Hierarchical clustering analysis was conducted for 1039 TSS200CGIs fully methylated in HCT116 using R function hclust.

DNA methylation status of the genes involved in cancer-related pathways, namely cell cycle regulation, WNT pathway, p53 pathway, and TGF-β pathway, were evaluated in their TSS200CGIs, except for *CDKN2A*. This lacked probes in its TSS200CGI, and its methylation was evaluated by a genomic block most 5′ of its exon 1, as described [36]. DNA methylation data was submitted to the Gene Expression Omnibus (GEO) database (accession no. GSE149255).

### Analysis of cell cycle

Cultured cells were washed with 1× PBS (-) and fixed with 70% ethanol. The fixed cells were treated with RNase A (Thermo Fisher Scientific, Waltham, MA) for 20 min, stained with 50 μg/ml propidium iodide (Sigma-Aldrich) for 10 min, and analyzed using an Attune NxT Flow Cytometer (Thermo Fisher Scientific).

## Supplementary information

**Additional file 1: Fig. S1.** DNA demethylation in surviving and dead cells by DAC treatment. (a) Experimental protocol of DAC treatment. HCT116 cells were seeded on day 0, treated with DAC on days 1 and 3, and placed in a fresh medium without DAC on day 5. Surviving cells were harvested at day 21, and floating cells, considered as dead cells, were harvested during days 5 to 21. (b) Analysis of DNA demethylation in surviving and dead cells. Both surviving and dead cells showed partial demethylation compared with mock-treated cells, but dead cells had 7.8% more demethylation than surviving cells based upon the mean decrease of the β values of all the genomic blocks. **Fig. S2.** Changes of DNA methylation levels in additional DAC-treated clones. Methylation levels were markedly reduced by DAC treatment. Methylation changes in the H3-01, H3-21, H3-22, H3-32, and H3-72 clones are shown. **Fig. S3.** Correlation analysis between the doubling time and the number of completely demethylated genes. No association was observed among the nine DAC-treated clones. **Fig. S4.** DNA demethylating and cytotoxic effects of DAC treatment. (a) Experimental protocol of DAC treatment. HCT116 cells were seeded on day 0, and treated with DAC on days 1 and 3. DNA methylation levels and cell number were analyzed on day 5. (b) Analysis of DNA demethylating effect. DNA methylation levels of *SFRP1*, *DCC*, and *ZNF229* were analyzed. The strongest DNA demethylation was observed with 0.5 μM of treatment. (c) Analysis of cytotoxic effect. Cell numbers were counted after DAC treatment. A dose-dependent cytotoxic effect was observed. **Table S1.** Overlap of completely demethylated genes (TSS200CGIs) among DAC-treated clones. **Tables S2.** Primers used for quantitative methylation-specific PCR.

## Data Availability

The datasets used in this study are available at the Gene Expression Omnibus (GEO) database (https://www.ncbi.nlm.nih.gov/geo/) with accession no. GSE149255.

## Abbreviations

DAC: Decitabine
CGI: CpG island
TSS: Transcription start site

## References

[1] Baylin SB, Jones PA (2011). A decade of exploring the cancer epigenome - biological and translational implications. Nat Rev Cancer.

[2] Esteller M (2007). Cancer epigenomics: DNA methylomes and histone-modification maps. Nat Rev Genet.

[3] Quintas-Cardama A, Santos FP, Garcia-Manero G (2010). Therapy with azanucleosides for myelodysplastic syndromes. Nat Rev Clin Oncol.

[4] Wouters BJ, Delwel R (2016). Epigenetics and approaches to targeted epigenetic therapy in acute myeloid leukemia. Blood..

[5] Pechalrieu D, Etievant C, Arimondo PB (2017). DNA methyltransferase inhibitors in cancer: from pharmacology to translational studies. Biochem Pharmacol.

[6] Linnekamp JF, Butter R, Spijker R, Medema JP, van Laarhoven HWM (2017). Clinical and biological effects of demethylating agents on solid tumours - a systematic review. Cancer Treat Rev.

[7] Nervi C, De Marinis E, Codacci-Pisanelli G (2015). Epigenetic treatment of solid tumours: a review of clinical trials. Clin Epigenetics.

[8] Juergens RA, Wrangle J, Vendetti FP, Murphy SC, Zhao M, Coleman B (2011). Combination epigenetic therapy has efficacy in patients with refractory advanced non-small cell lung cancer. Cancer Discov.

[9] Matei D, Fang F, Shen C, Schilder J, Arnold A, Zeng Y (2012). Epigenetic resensitization to platinum in ovarian cancer. Cancer Res.

[10] Mei Q, Chen M, Lu X, Li X, Duan F, Wang M (2015). An open-label, single-arm, phase I/II study of lower-dose decitabine based therapy in patients with advanced hepatocellular carcinoma. Oncotarget..

[11] Chen M, Nie J, Liu Y, Li X, Zhang Y, Brock MV (2018). Phase Ib/II study of safety and efficacy of low-dose decitabine-primed chemoimmunotherapy in patients with drug-resistant relapsed/refractory alimentary tract cancer. Int J Cancer.

[12] Momparler RL (2005). Epigenetic therapy of cancer with 5-aza-2′-deoxycytidine (decitabine). Semin Oncol.

[13] Issa JP, Garcia-Manero G, Giles FJ, Mannari R, Thomas D, Faderl S (2004). Phase 1 study of low-dose prolonged exposure schedules of the hypomethylating agent 5-aza-2′-deoxycytidine (decitabine) in hematopoietic malignancies. Blood..

[14] Issa JP, Gharibyan V, Cortes J, Jelinek J, Morris G, Verstovsek S (2005). Phase II study of low-dose decitabine in patients with chronic myelogenous leukemia resistant to imatinib mesylate. J Clin Oncol.

[15] Kantarjian H, Oki Y, Garcia-Manero G, Huang X, O'Brien S, Cortes J (2007). Results of a randomized study of 3 schedules of low-dose decitabine in higher-risk myelodysplastic syndrome and chronic myelomonocytic leukemia. Blood..

[16] Bender CM, Pao MM, Jones PA (1998). Inhibition of DNA methylation by 5-aza-2′-deoxycytidine suppresses the growth of human tumor cell lines. Cancer Res.

[17] Tsai HC, Li H, Van Neste L, Cai Y, Robert C, Rassool FV (2012). Transient low doses of DNA-demethylating agents exert durable antitumor effects on hematological and epithelial tumor cells. Cancer Cell.

[18] Chiappinelli KB, Strissel PL, Desrichard A, Li H, Henke C, Akman B (2015). Inhibiting DNA methylation causes an interferon response in cancer via dsRNA including endogenous retroviruses. Cell..

[19] Roulois D, Loo Yau H, Singhania R, Wang Y, Danesh A, Shen SY (2015). DNA-demethylating agents target colorectal cancer cells by inducing viral mimicry by endogenous transcripts. Cell..

[20] Maeda M, Takeshima H, Iida N, Hattori N, Yamashita S, Moro H (2020). Cancer cell niche factors secreted from cancer-associated fibroblast by loss of H3K27me3. Gut..

[21] Pidsley R, Lawrence MG, Zotenko E, Niranjan B, Statham A, Song J (2018). Enduring epigenetic landmarks define the cancer microenvironment. Genome Res.

[22] Lu Z, Zou J, Li S, Topper MJ, Tao Y, Zhang H (2020). Epigenetic therapy inhibits metastases by disrupting premetastatic niches. Nature..

[23] Brandes JC, van Engeland M, Wouters KA, Weijenberg MP, Herman JG (2005). CHFR promoter hypermethylation in colon cancer correlates with the microsatellite instability phenotype. Carcinogenesis..

[24] Kong X, Chen J, Xie W, Brown SM, Cai Y, Wu K (2019). Defining UHRF1 domains that support maintenance of human colon cancer DNA methylation and oncogenic properties. Cancer Cell.

[25] Suzuki H, Gabrielson E, Chen W, Anbazhagan R, van Engeland M, Weijenberg MP (2002). A genomic screen for genes upregulated by demethylation and histone deacetylase inhibition in human colorectal cancer. Nat Genet.

[26] Alcorta DA, Xiong Y, Phelps D, Hannon G, Beach D, Barrett JC (1996). Involvement of the cyclin-dependent kinase inhibitor p16 (INK4a) in replicative senescence of normal human fibroblasts. Proc Natl Acad Sci U S A.

[27] Hara E, Smith R, Parry D, Tahara H, Stone S, Peters G (1996). Regulation of p16CDKN2 expression and its implications for cell immortalization and senescence. Mol Cell Biol.

[28] Schneider BJ, Shah MA, Klute K, Ocean A, Popa E, Altorki N (2017). Phase I study of epigenetic priming with azacitidine prior to standard neoadjuvant chemotherapy for patients with resectable gastric and esophageal adenocarcinoma: evidence of tumor hypomethylation as an indicator of major histopathologic response. Clin Cancer Res.

[29] Singal R, Ramachandran K, Gordian E, Quintero C, Zhao W, Reis IM (2015). Phase I/II study of azacitidine, docetaxel, and prednisone in patients with metastatic castration-resistant prostate cancer previously treated with docetaxel-based therapy. Clin Genitourin Cancer.

[30] Tawbi HA, Beumer JH, Tarhini AA, Moschos S, Buch SC, Egorin MJ (2013). Safety and efficacy of decitabine in combination with temozolomide in metastatic melanoma: a phase I/II study and pharmacokinetic analysis. Ann Oncol.

[31] Bauman J, Verschraegen C, Belinsky S, Muller C, Rutledge T, Fekrazad M (2012). A phase I study of 5-azacytidine and erlotinib in advanced solid tumor malignancies. Cancer Chemother Pharmacol.

[32] Garrido-Laguna I, McGregor KA, Wade M, Weis J, Gilcrease W, Burr L (2013). A phase I/II study of decitabine in combination with panitumumab in patients with wild-type (wt) KRAS metastatic colorectal cancer. Investig New Drugs.

[33] Becht E, McInnes L, Healy J, Dutertre CA, Kwok IWH, Ng LG, et al. Dimensionality reduction for visualizing single-cell data using UMAP. Nat Biotechnol. 2018. 10.1038/nbt.4314 30531897

[34] Takeshima H, Niwa T, Takahashi T, Wakabayashi M, Yamashita S, Ando T (2015). Frequent involvement of chromatin remodeler alterations in gastric field cancerization. Cancer Lett.

[35] Iida N, Okuda Y, Ogasawara O, Yamashita S, Takeshima H, Ushijima T (2018). MACON: a web tool for computing DNA methylation data obtained by the Illumina Infinium Human DNA methylation BeadArray. Epigenomics..

[36] Yoda Y, Takeshima H, Niwa T, Kim JG, Ando T, Kushima R (2015). Integrated analysis of cancer-related pathways affected by genetic and epigenetic alterations in gastric cancer. Gastric Cancer.

