# Supplementary material for: Low-dose DNA demethylating therapy induces reprogramming of diverse cancer-related pathways at the single-cell level
Source: Clin Epigenetics. 2020 Sep 21;12:142. doi: 10.1186/s13148-020-00937-y (PMC7507826; doi:10.1186/s13148-020-00937-y)
Supplement: Supplementary file 1 — Additional file 1: Fig. S1. DNA demethylation in surviving and dead cells by DAC treatment. (a) Experimental protocol of DAC treatment. HCT116 cells were seeded on day 0, treated with DAC on days 1 and 3, and placed in a fresh medium without DAC on day 5. Surviving cells were harvested at day 21, and floating cells, considered as dead cells, were harvested during days 5 to 21. (b) Analysis of DNA demethylation in surviving and dead cells. Both surviving and dead cells showed partial demethylation compared with mock-treated cells, but dead cells had 7.8% more demethylation than surviving cells based upon the mean decrease of the β values of all the genomic blocks. Fig. S2. Changes of DNA methylation levels in additional DAC-treated clones. Methylation levels were markedly reduced by DAC treatment. Methylation changes in the H3-01, H3-21, H3-22, H3-32, and H3-72 clones are shown. Fig. S3. Correlation analysis between the doubling time and the number of completely demethylated genes. No association was observed among the nine DAC-treated clones. Fig. S4. DNA demethylating and cytotoxic effects of DAC treatment. (a) Experimental protocol of DAC treatment. HCT116 cells were seeded on day 0, and treated with DAC on days 1 and 3. DNA methylation levels and cell number were analyzed on day 5. (b) Analysis of DNA demethylating effect. DNA methylation levels of SFRP1, DCC, and ZNF229 were analyzed. The strongest DNA demethylation was observed with 0.5 μM of treatment. (c) Analysis of cytotoxic effect. Cell numbers were counted after DAC treatment. A dose-dependent cytotoxic effect was observed. Table S1. Overlap of completely demethylated genes (TSS200CGIs) among DAC-treated clones. Tables S2. Primers used for quantitative methylation-specific PCR. [file 13148_2020_937_MOESM1_ESM.docx]

# Supplementary Information

**Low-dose DNA demethylating therapy induces reprogramming of diverse cancer-related pathways at the single-cell level**

Hideyuki Takeshima, Yukie Yoda, Mika Wakabayashi, Naoko Hattori, Satoshi Yamashita, and Toshikazu Ushijima

# Supplementary Method

## DNA methylation analysis of specific genes

DNA methylation of specific genes was analyzed by quantitative methylation-specific PCR (qMSP) as described [1] using primers listed in Table S2. The DNA methylation level was calculated as the fraction of methylated molecules in both methylated and unmethylated molecules.


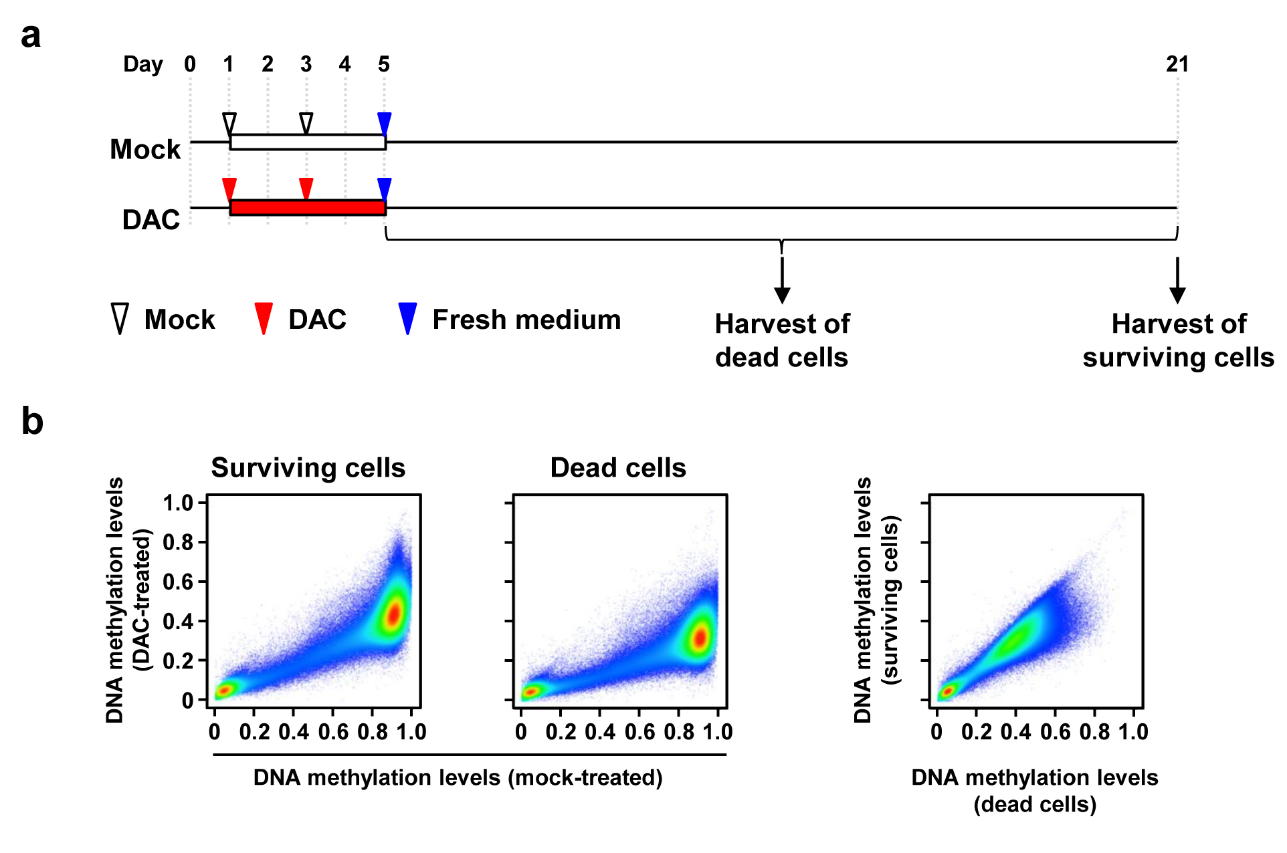


## Fig. S1.

DNA demethylation in surviving and dead cells by DAC treatment. (a) Experimental protocol of DAC treatment. HCT116 cells were seeded on day 0, treated with DAC on days 1 and 3, and placed in a fresh medium without DAC on day 5. Surviving cells were harvested at day 21, and floating cells, considered as dead cells, were harvested during days 5 to 21. (b) Analysis of DNA demethylation in surviving and dead cells. Both surviving and dead cells showed partial demethylation compared with mock-treated cells, but dead cells had 7.8% more demethylation than surviving cells based upon the mean decrease of the β values of all the genomic blocks.


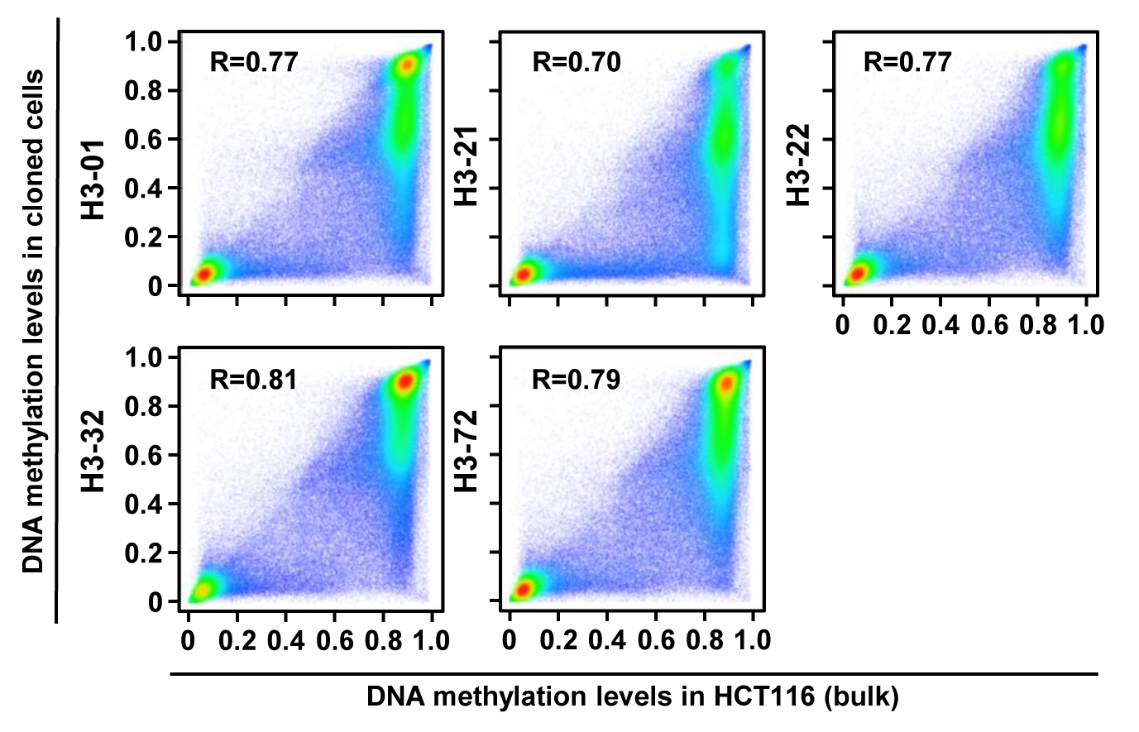


## Fig. S2.

Changes of DNA methylation levels in additional DAC-treated clones. Methylation levels were markedly reduced by DAC treatment. Methylation changes in the H3-01, H3-21, H3-22, H3-32, and H3-72 clones are shown.


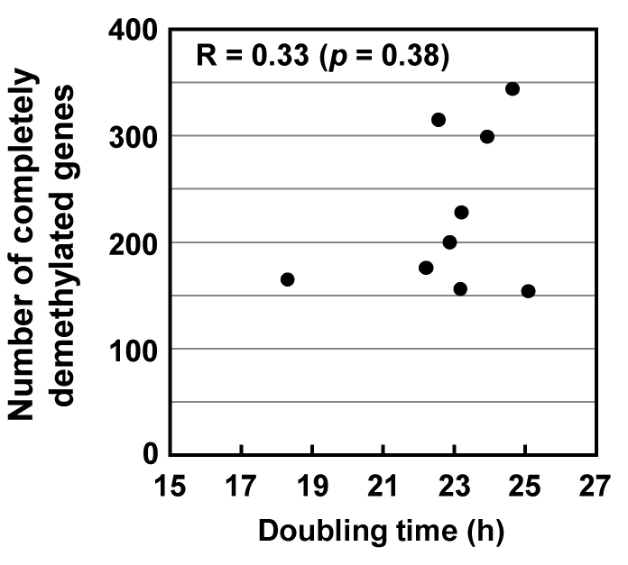


## Fig. S3.

Correlation analysis between the doubling time and the number of completely demethylated genes. No association was observed among the nine DAC-treated clones.


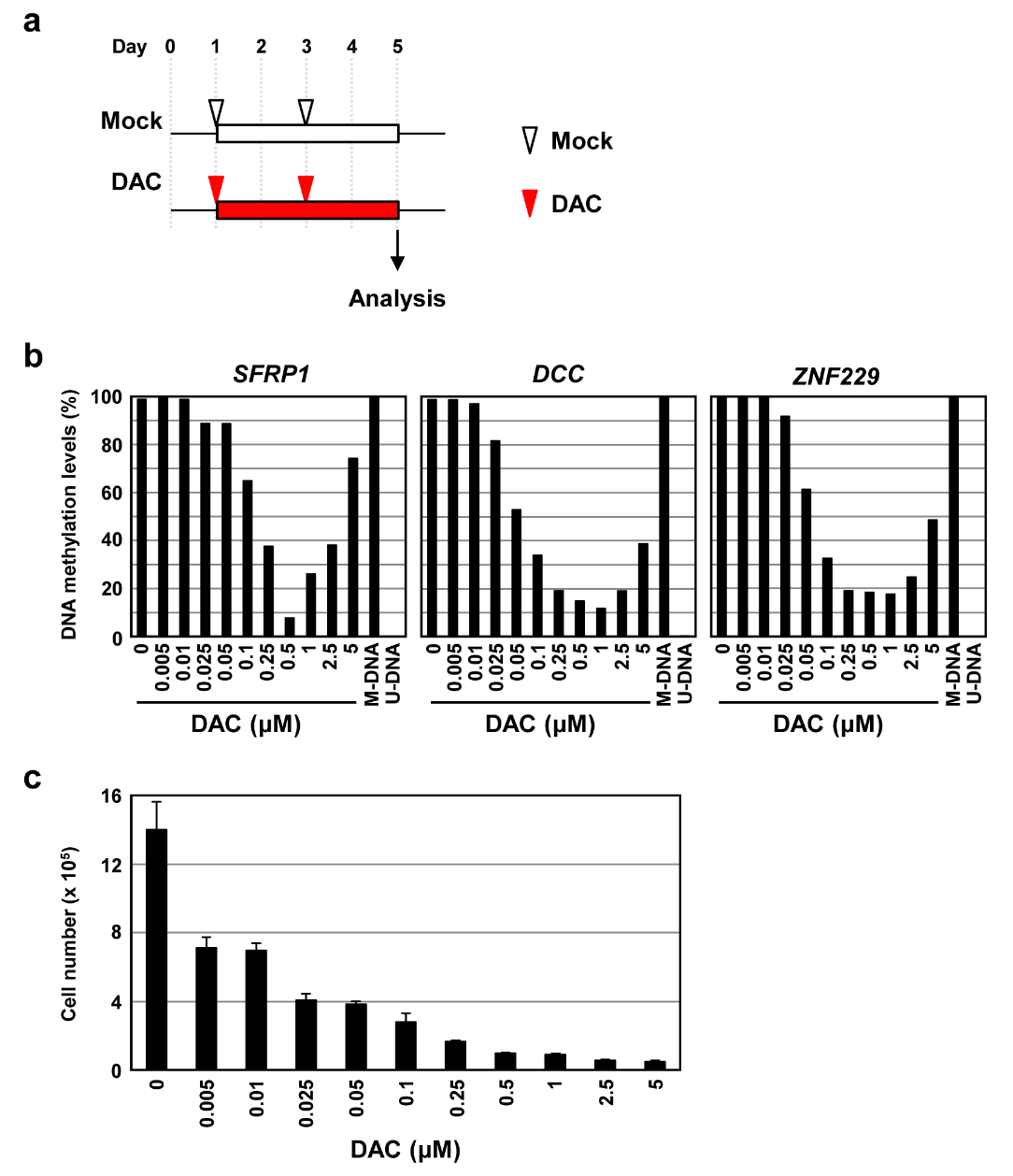


## Fig. S4.

DNA demethylating and cytotoxic effects of DAC treatment. (a) Experimental protocol of DAC treatment. HCT116 cells were seeded on day 0, and treated with DAC on days 1 and 3. DNA methylation levels and cell number were analyzed on day 5. (b) Analysis of DNA demethylating effect. DNA methylation levels of *SFRP1*, *DCC*, and *ZNF229* were analyzed. The strongest DNA demethylation was observed with 0.5 μM of treatment. (c) Analysis of cytotoxic effect. Cell numbers were counted after DAC treatment. A dose-dependent cytotoxic effect was observed.

|  |  | H3-01 | H3-21 | H3-22 | H3-25 | H3-30 | H3-32 | H3-33 | H3-71 | H3-72 |
| --- | --- | --- | --- | --- | --- | --- | --- | --- | --- | --- |
| Total number of completely demethylated genes |  | 191 | 264 | 133 | 257 | 169 | 126 | 132 | 293 | 135 |
| Overlapped genes with |  |  |  |  |  |  |  |  |  |  |
| H3-01 |  | - |  |  |  |  |  |  |  |  |
| H3-21 |  | 83 (43.5%) | - |  |  |  |  |  |  |  |
| H3-22 |  | 58 (30.4%) | 63 (23.9%) | - |  |  |  |  |  |  |
| H3-25 |  | 59 (30.9%) | 72 (27.3%) | 56 (42.1%) | - |  |  |  |  |  |
| H3-30 |  | 41 (21.5%) | 47 (17.8%) | 37 (27.8%) | 71 (27.6%) | - |  |  |  |  |
| H3-32 |  | 31 (16.2%) | 49 (18.6%) | 25 (18.8%) | 44 (17.1%) | 29 (17.2%) | - |  |  |  |
| H3-33 |  | 47 (24.6%) | 40 (15.2%) | 34 (25.6%) | 49 (19.1%) | 32 (18.9%) | 35 (27.8%) | - |  |  |
| H3-71 |  | 84 (44%) | 125 (47.3%) | 77 (57.9%) | 139 (54.1%) | 71 (42%) | 66 (52.4%) | 61 (46.2%) | - |  |
| H3-72 |  | 54 (28.3%) | 51 (19.3%) | 29 (21.8%) | 59 (23%) | 41 (24.3%) | 32 (25.4%) | 36 (27.3%) | 62 (21.2%) | - |

Table S1. Overlap of completely demethylated genes (TSS200CGIs) among DAC-treated clones.

| Table S2. Primers used for quantitative methylation-specific PCR. | | | |  |
| --- | --- | --- | --- | --- |
| Gene | Type |  | Forward primer sequence | Reverse primer sequence |
| *DCC* | M |  | ATTTTTAGACGTTTAGGATGTTC | CCTAATCTACGTATCAATACG |
| *DCC* | U |  | TGAAGGTTGAGTGTTAAATATGGT | ACCTAATCTACATATCAATACA |
| *SFRP1* | M |  | TTTAGTAAATCGAATTCGTTCGC | ATACGCGAAACTCCTACGACGG |
| *SFRP1* | U |  | GGTTTGGTTGTAGGAGTTTTGT | CCCCAACTCCCAAAAATACAACA |
| *ZNF229* | M |  | TGGACGCGTATTTACGATATTATC | CGAAAACGAAAATAATCGACTCG |
| *ZNF229* | U |  | AGGTGGATGTGTATTTATGATATTATT | CACCAAAAACAAAAATAATCAACTCA |
| M, primers specific to methylated DNA; U, primers specific to unmethylated DNA. | | | | |

# Supplementary Reference

1. Takeshima H, Niwa T, Takahashi T, Wakabayashi M, Yamashita S, Ando T, et al. Frequent involvement of chromatin remodeler alterations in gastric field cancerization. Cancer Lett. 2015;357:328-338.
